# A combined experimental-numerical approach for determining mechanical properties of aluminum subjects to nanoindentation

**DOI:** 10.1038/srep15072

**Published:** 2015-10-14

**Authors:** Mao Liu, Cheng Lu, Kiet Anh Tieu, Ching-Tun Peng, Charlie Kong

**Affiliations:** 1 School of Mechanical, Materials and Mechatronic Engineering, University of Wollongong, Wollongong, NSW 2522, Australia; 2 Electron Microscope Unit, The University of New South Wales, Sydney, NSW 2052, Australia

## Abstract

A crystal plasticity finite element method (CPFEM) model has been developed to investigate the mechanical properties and micro-texture evolution of single-crystal aluminum induced by a sharp Berkovich indenter. The load-displacement curves, pile-up patterns and lattice rotation angles from simulation are consistent with the experimental results. The pile-up phenomenon and lattice rotation have been discussed based on the theory of crystal plasticity. In addition, a polycrystal tensile CPFEM model has been established to explore the relationship between indentation hardness and yield stress. The elastic constraint factor C is slightly larger than conventional value 3 due to the strain hardening.

Indentation is widely used as a testing method to determine the mechanical properties of materials. The penetration depth in conventional indentation tests has a length scale in terms of microns or millimetres. Since mid-1970s, the indentation technique was applied to measure the hardness of small volumes of material, such as thin film. Nowadays, numerous publications report scanning indentation depths of 10 to 50 nm in particular and <200 nm in general^1^,^2^,^3^,^4^,^5^. This is what makes the technique known as nanoindentation.

The single-crystal metals are mostly studied because of their extensive applications. The dependence of nanoindentation pile-up patterns and micro-textures on the crystallographic orientation were studied by Wang *et al.*^6^ using high purity single-crystal copper (Cu) with three different initial orientations. Four-, two-, and six-fold symmetrical pile-up patterns were captured on the surface of (001), (011) and (111) oriented single crystal, respectively. Lloyd^7^ and his colleagues combined nanoindentation and transmission electron microscopy (TEM) to survey the deformation behaviour in a range of single-crystal materials with different resistances to dislocation flow. It was found that the shear band spacing increased with increasing distance from the indent tip, and the spacing on the steep side of the indent was slightly smaller for the large load. Lloyd^8^ concluded that the increase of the shear band spacing at distance far away from the indenter tip indicated that there was a limit to the amount of displacement occurring through any shear band due to strain hardening. Zaafarani *et al.*^9^ investigated texture and microstructure below a conical nano-indent in a (111) oriented single-crystal Cu using the three-dimensional (3D) electron backscatter diffraction (EBSD). The tests were performed using a joint high-resolution field emission scanning electron microscopy/EBSD set-up coupled with serial sectioning in a focused ion beam (FIB) system in the form of a cross-beam 3D crystal orientation microscope. The EBSD testings conducted in sets of one cross-section planes exhibited a pronounced deformation-induced 3D patterning of the lattice rotations below and around the indent.

The finite element method (FEM) modelling is another common method to investigate deformation mechanism of the materials discussed in aforementioned studies. Lee and Kobayashi^10^ were the first to conduct the FEM simulation of indentation in 1969. Plane strain and axisymmetric flat punch indentation were simulated to study the development of the plastic zone, the load-displacement relationships, and the stress and strain distributions during continuous loading, taking into account the changes of the punch friction and specimen dimensions. However, problems such as the accuracy of the solutions and the efficiency of the computation still exist. Bhattacharya and Nix^11^ performed elasto-plastic FEM simulations of nanoindentation using conical indenter to study the elastic and plastic properties of materials at a sub-micro scale under the conditions of frictionless and completely adhesive contact. The simulated load-displacement curves for nickel and silicon were consistent with experimental results. Hence, it was concluded that the FEM is suitable to simulate nanoindentation behaviour at a sub-micro scale for different types of materials.

However, the evolution of crystallographic texture and grain lattice rotation under the indentation are not well understood. Such analysis must be done through the crystal plasticity FEM (CPFEM) simulation. Only a few experimental studies have addressed the relationship between indentation and deformation-induced lattice rotations in the vicinity of an indent^9^ (as shown in Table 1).

Casals and Forest^12^ investigated the anisotropy in the contact response of face-centered cubic (FCC) and hexagonal close packed (HCP) single crystals by simulating the spherical indentation experiments of bulk single crystals and thin films on hard substrates. Their simulations revealed that the plastic zone beneath the indenter preferentially grew along the slip system directions. Consequently, in coated thin film systems, a prominent localization of plastic deformation occurred at those specific regions where the slip system directions and the substrate intersect. Meanwhile, these specific areas are prone to crack nucleation due to accumulative plastic damage. Therefore, the identification of these areas was meaningful for the prediction of potential delamination and failure of the coatings. Casals *et al.*^13^ used three-dimensional CPFEM simulations to examine Vickers and Berkovich indentation experiments of strain-hardened Cu. The results showed that the simulation was in a good agreement with experimental observations in terms of hardness, load-displacement curves, material pile-up and sink-in development at the contact boundary. Alcala *et al.*^14^ analysed Vickers and Berkovich indentation behaviour via extensive CPFEM simulation by recourse to the Bassani and Wu^15^ hardening model for pure FCC crystals. The simulated results have been used to illustrate the impact of the crystallographic orientation. It is clear that the irregular appearance of pyramidal indentations was governed by the crystallography of FCC crystals on the indented surface. Zaafarani *et al.*^9^ carried out the 3D elastic-viscoplastic CPFEM simulations with the same geometry of indenter and boundary conditions as those from experiments. Their simulations predicted a similar pattern for the absolute orientation changes as the experiments. However, it was found that the simulations overestimated the magnitude of the rotation field tangent to the indenter relative to that directly below the indenter tip. The reason was then found to be due to the edge effects at the contact zone and milling-induced curvature caused by ion beam so that no complete EBSD mapping could be made up to the actual contact interface^16^. Eidel^17^ simulated pyramidal micro-indentation on the (001) surface of single-crystal Ni-base superalloy with three different azimuthal orientations of the pyramidal indenter. The numerical pile-up patterns were then compared with the experimental results. It was found that the resultant material pile-up was insensitive to different azimuthal orientations of the pyramidal indenter. This could be due to the pile-up which is solely determined by crystallographic processes rather than by the stress distribution pattern, induced under the non-isotropic pyramidal indenter. He also found that the pile-up was independent of the indenter shape (sphere or pyramid) and the elastic anisotropy of measured materials. It further confirmed that only the geometry of the slip systems in the (001) oriented crystal governed pile-up. On the other hand, the stress concentrations introduced by the different indenter shapes, the different azimuthal orientations of a pyramidal indenter and the characteristics of the elasticity law have insignificant influence. However, the further investigation is needed to understand the correlation between the slip systems and the pile-up patterns. Liu *et al.*^18^ performed CPFEM simulation on (001), (010) and (111) initially oriented surfaces of the single-crystal Cu with a spherical indenter. Their simulation is consistent with experiment observations in terms of mechanical properties of single-crystal Cu.

Most of the aforementioned literatures^6^,^9^,^16^,^17^,^18^,^19^ associated with nanoindentation modelling adopted the hardening rule originally proposed by Brown *et al.*^20^ and Kalidindi^21^, which is a form of the single slip hardening rate. Lin and Havner^22^ comparatively studied five hardening rules and they concluded that Bassani and Wu hardening model is the best predictor of experiments among the five theories when carrying out crystal plasticity modelling of torsion. Besides, most of the reported nanoindentation simulations adopted spherical indenter as it is really difficult to get convergence when using a sharp Berkovich indenter during modelling process, while adopting the user material subroutine (UMAT). Nevertheless, most of studies have shown that they are only capable of predicting either mechanical properties (e.g. P-h curve) or micro-texture (e.g. lattice rotation angle) of nanoindentation induced single-crystal materials accurately. In this study, a CPFEM model coupled with Bassani-Wu hardening model, has been developed to investigate the mechanical properties and micro-texture evolution of single-crystal aluminium (Al) with three well-defined initial orientations undergoing nanoindentation via a Berkovich indenter. The load-displacement curves, pile-up patterns, elastic modulus, hardness and lattice rotation angles are compared with the experimental results from nanoindentation tests. In addition, a poly-crystal CPFEM tensile model has also been established to study the relationship between indentation hardness and yield stress.

## Method

### Crystal plasticity finite element method modelling

The crystal plasticity constitutive model (as shown in the supplementary material) is implemented into the implicit finite element code ABAQUS/Standard by using the UMAT which is able to provide the material Jacobian matrix, ∂Δ*σ*/∂Δ*ε*, for the constitutive model and to update the stresses and the solution dependent state variables. In this study, the UMAT framework initially developed by Huang^23^ and the formulations established by Bassani and Wu^15^ as the hardening model are adopted.

**Figure 1:**
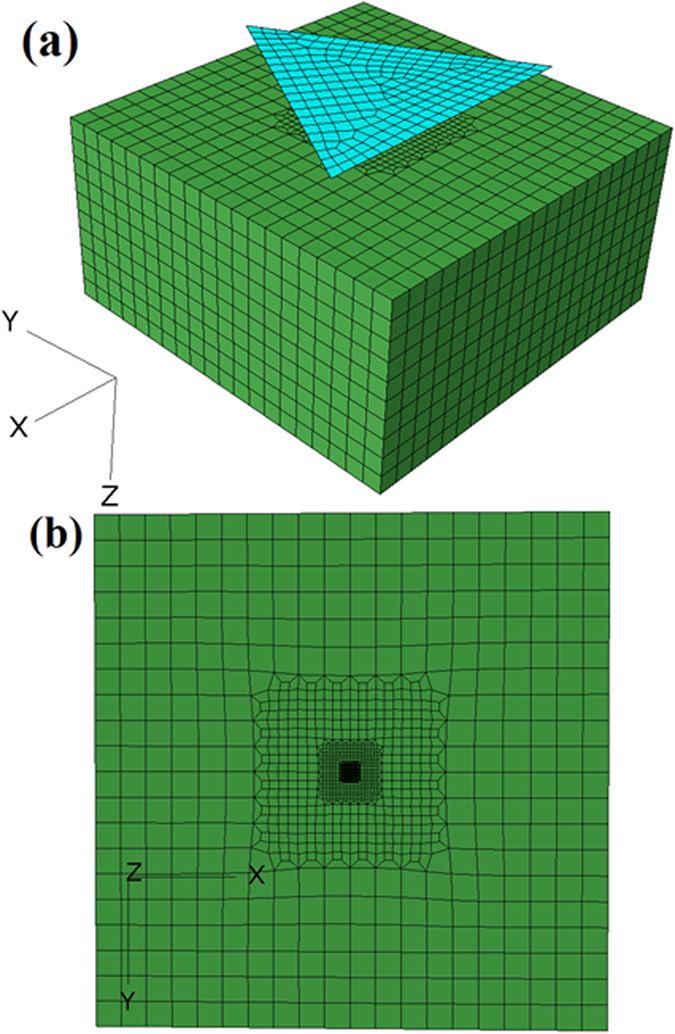
3D nanoindentation model setup.

The commercial software Abaqus6.9 is used to simulate the deformation procedure of nanoindentation. A 3D model is established to describe the mechanical behaviour of single-crystal Al induced by nanoindentation, which is shown in Fig. 1.

The indenter shown in Fig. 1 is a Berkovich indenter with a 200 nm radius round tip. 13024 eight-node brick elements and 14463 nodes with reduced integration (element id: C3D8R) are used in the CPFEM model. A refined mesh is generated in the contact area (Fig. 1b) directly underneath the indenter in order to obtain an accurate contact solution while a coarser mesh was created in the rest region to decrease the elements number in the model, and thus, reduces the computational time. The importance of having an appropriate mesh density in the contact area has been proposed in ref. 18,19. The size of the smallest element was about 0.1 μm in all three directions. The *x*, *y* and *z* coordinates represent the rolling direction (RD), transverse direction (TD) and normal direction (ND) respectively. In this model, the initial specific orientations of z-plane are namely the (001), (101), and (111) slip planes. The tangent stiffness matrix (Jacobian matrix) is not symmetrical as the latent hardness is considered. Therefore, it must be declared “unsymm” in the input file at the user material card.

The dimensions of the workpiece in the FEM model are given as 60 × 60 × 30 μm. The height of the workpiece is much larger than the maximum indentation depth (1 μm), so as to avoid the influence from the workpiece^24^. In addition, the length and the width must also be large enough to ensure that the stress contour will never reach the boundaries of the workpiece.

The micro-scale mechanical behaviour of single-crystal Al is investigated via nanoindentation. Liu *et al.*^19^ have showed that the coefficient of friction (COF) has an insignificant effect on both the indentation depth and the load-displacement curve. Therefore, a frictionless contact pair is defined by two contact surfaces with associated nodes between the indenter and workpiece. The time step increment is also set for the convergence of modelling. In this study, a fixed time step increment of 0.01 s is adopted in the simulation. The total time step increments of 24,062 are performed throughout the whole simulation, including contact, loading, and unloading.

Franciosi *et al.*^25^ and Lu *et al.*^26^ have reported the factor *f*_*αβ*_ for Al can be chosen as: *α*_1_ = *α*_2_ = *α*_3_ = 1.75, *α*_4_ = 2 and *α*_5_ = 2.25. Other material parameters in the hardening models (Eqs. (62–66) in supplementary materials) are listed in Table 2. All of these parameters are evaluated by fitting the simulated stress-strain curve with the experimental results of single- crystal Al under plane strain compression^27^,^28^. Aluminium has an FCC structure with elastic moduli C_11_ = 112,000 MPa, C_12_ = 66,000 MPa and C_44_ = 28,000 MPa. There is one set of slip systems for FCC metals, which is {111} <110>. There are a total of 12 different slip systems (as shown in Table 3). In the deformed single-crystal Al, slips occur on the {111} planes and in the <110> directions. All of aforementioned parameters have been validated in the simulations of nanoindentation, rolling, tensile and Equal channel angular processing (ECAP) deformation^26^,^29^,^30^,^31^,^32^.

### Experimental setup

#### Material and sample preparations

The materials used in the nanoindentation tests was single-crystal Al disks with a purity of 99.9999% wt.%, provided by MaTecK. The detailed information of the raw materials is shown in Table 4.

The single-crystal Al disk samples with three different orientations are prepared for the nanoindentation tests. The diameter and thickness of the samples are 15 mm and 2 mm respectively. The three orientations are (100), (101) and (111) and they are parallel to the surface of the sample. All the samples are electro-polished before indentation. The roughness of polished surface is less than 10 nm, measured by an atomic force microscope (AFM). The EBSD technique is then employed to measure the distribution of the crystallographic orientation after the nanoindentation tests.

#### Indentation tests

Nanoindentation tests are conducted on the single-crystal Al samples with (001), (101) and (111) orientations. The experimental results are subsequently compared with numerical simulations based on the CPFEM effort. The IBIS nanoindentation system (Model A) with a maximum load of 100 mN and a maximum indentation displacement of 5 μm is used to conduct the tests. The displacement resolution is 0.05 nm and the load resolution is 75 nN. All nanoindentation tests are carried out in air at room temperature and during the night, so as to achieve a thermal drift of 0.05 nm/s and reduce other effects. 200 data points are recorded automatically for the indentation load and displacement during loading and unloading process respectively[Fig f2].

The three single-crystal Al samples with (001), (101) and (111) oriented surfaces are electro-polished for nanoindentation tests. An EBSD pattern is then used to check the purity of the sample and no microstructural distortions or disorientation of the crystal in the surface layer of the sample is detected in terms of the IPF (inverse pole figure) mapping (às shown in Fig. 3). Each sample is mounted separately on the smooth surface of an Al cylinder with a thin layer of epoxy glue. Nanoindentation tests are conducted using a Berkovich diamond indenter with a radius of 200 nm. A 6 × 6 indentation matrices are conducted on each individual sample. All the indents are located in the middle of the sample and far away from the edge of the sample in order to avoid edge effects. Meanwhile, the distance between each indent is set as 200 μm, which is 25 times more than the indent impression diameter and thus, any mutual interaction can be avoided. After the nanoindentation tests, the EBSD is used to scan the indented surface to obtain an accurate orientation of the indent. During the scanning, the indented surface is set to be perpendicular to the ND and one base edge of one chosen indent is set to be parallel to the RD. The Euler angles from the EBSD test then is subsequently converted to Miller indices, which are then substituted into UMAT for simulation. The AFM is also used to obtain the surface topographies and the pile-up profiles by scanning the indented surfaces. The sample with (101) initial orientation is subjected to the focused ion beam (FIB) test in order to obtain the cross-sectional sample of the indent. Selected area diffraction (SAED) tests were then conducted on the FIB sample to analyse the lattice rotation angles using the SAED patterns. All the experimental data are subsequently compared with the numerical results.

## Results

### Mechanical properties

**Figure 2 f2:**
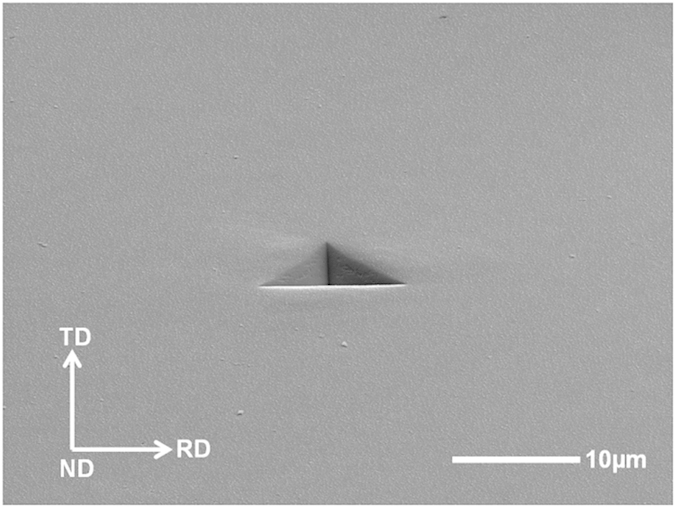
The selected indent for EBSD scanning.

Figure 2 shows the selected indent for EBSD scanning. The indented surface is set to be perpendicular to the ND and one edge of the indent is set to be parallel to the RD.

**Figure 3:**
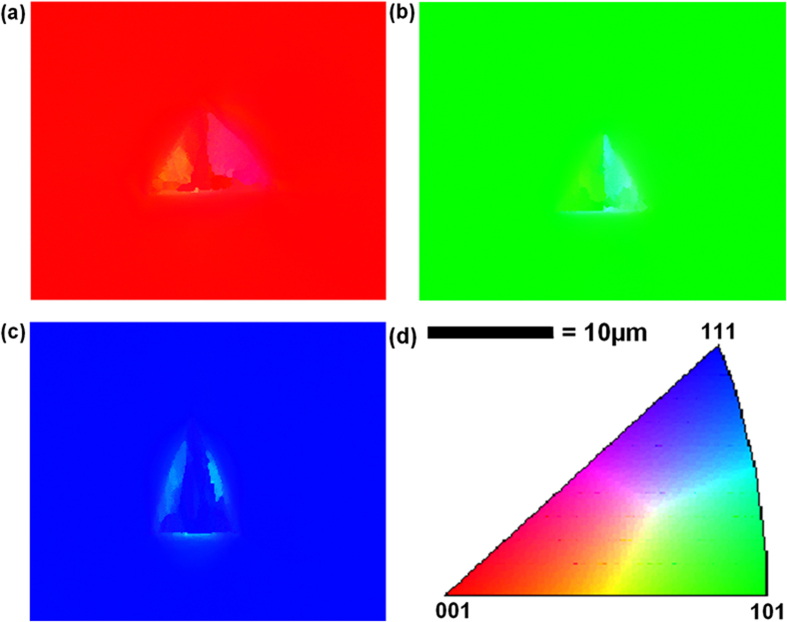
EBSD IPF mapping of the selected indent on different initial oriented surfaces: (**a**) (001), (**b**) (101) and (**c**) (111) surfaces.

Figure 3 shows the IPF mapping of the selected indents on the three different initial oriented surfaces. It is apparent that the single-crystal Al samples used in this study are of high quality and purity. All the Euler angles from the scanning can be converted to Miller indices using the following equation.


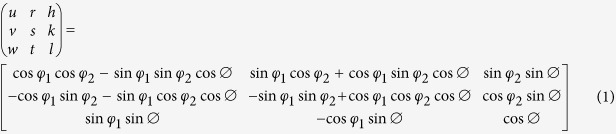


**Figure 4:**
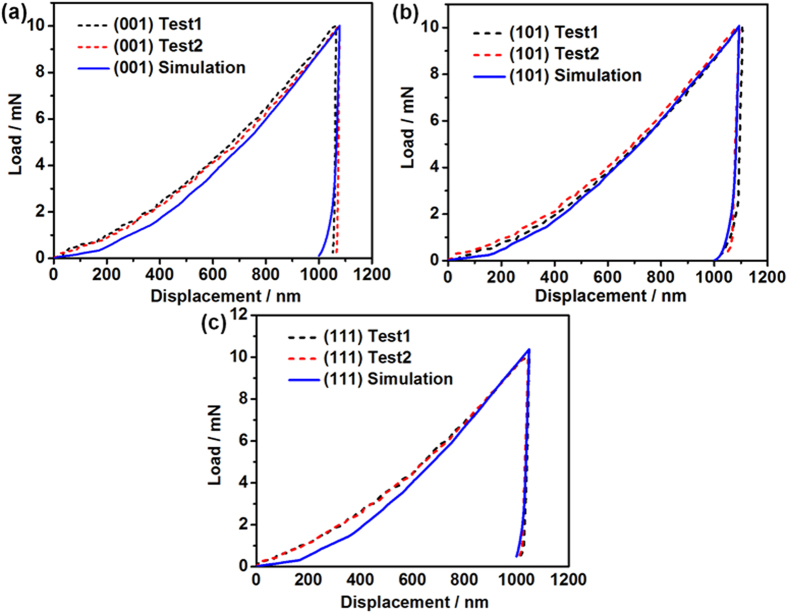
Comparisons between numerical and experimental load-displacement curves for single-crystal Al samples: (**a**) (001), (**b**) (101) and (**c**) (111) surfaces.

Figure 4 shows the comparisons between numerical and experimental load-displacement curves for single-crystal Al on the three different oriented surfaces. Many studies have failed to show any correlation between numerical and experimental load-displacement curves of single-crystal Al for all three orientations, especially when the Berkovich indenter is used. Liu *et al.*^18^ have compared the experimental results with the simulated load-displacement curves on the (001), (011) and (111) oriented single-crystal Cu using ABAQUS with a user-defined material subroutine VUMAT. A spherical indenter is used in their study. Wang *et al.*^6^ implemented the constitutive model for single crystalline Cu and the implicit time-integration procedure proposed by Kalidindi *et al.*^21^ into the commercial finite element code MARC by means of the user defined material subroutine HYPELA2 to perform the nanoindentation simulation. They indicated that the experimental and simulated load-displacement curves are generally very difficult to compare, and thus they presented the comparison of the pile-up patterns instead.

**Figure 5:**
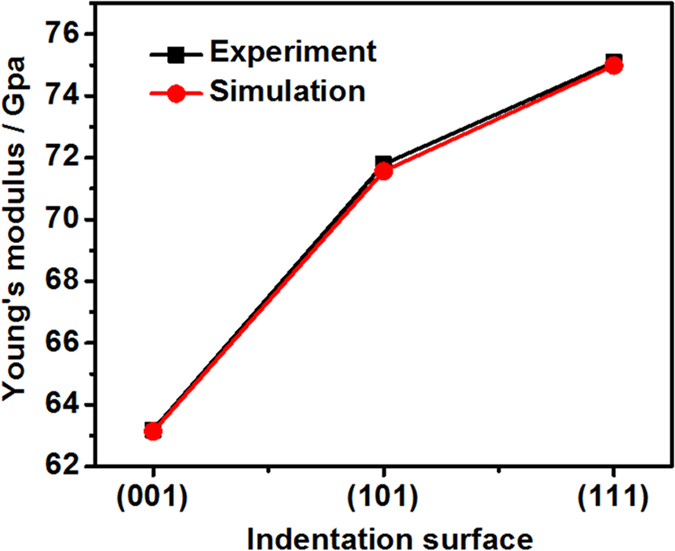
Comparisons of Young’s modulus between numerical and experimental results for single-crystal Al samples.

Figure 5 shows the comparison of Young’s modulus between the numerical and experimental results. There is a good correlation between the simulation and experimental results (as shown in Table 5). The simulated Young’s modulus can be calculated by the equations described in ref. 31. The measured Young’s modulus for the (001), (101) and (111) crystals are 63.18 GPa, 71.79 GPa and 75.09 GPa respectively. The Young’s modulus of the (111) crystal is ~15.9% larger than that of the (001) crystal. Wang and Lu^33^ found that the difference in the measured Young’s modulus between the (111) and (001) Cu crystals is ~20%. The Young’s moduli for three different low-index directions can be calculated based on the equations described in the ref. 34 via three elastic moduli *C*_*11*_, *C*_*12*_ and *C*_*44*_, which are E_001_ = 63.14 GPa, E_101_ = 71.56 GPa and E_111_ = 74.98 GPa respectively. It suggests that both experimentally measured and simulated Young’s moduli are in reasonable agreements with those from three different low-index directions.

**Figure 6:**
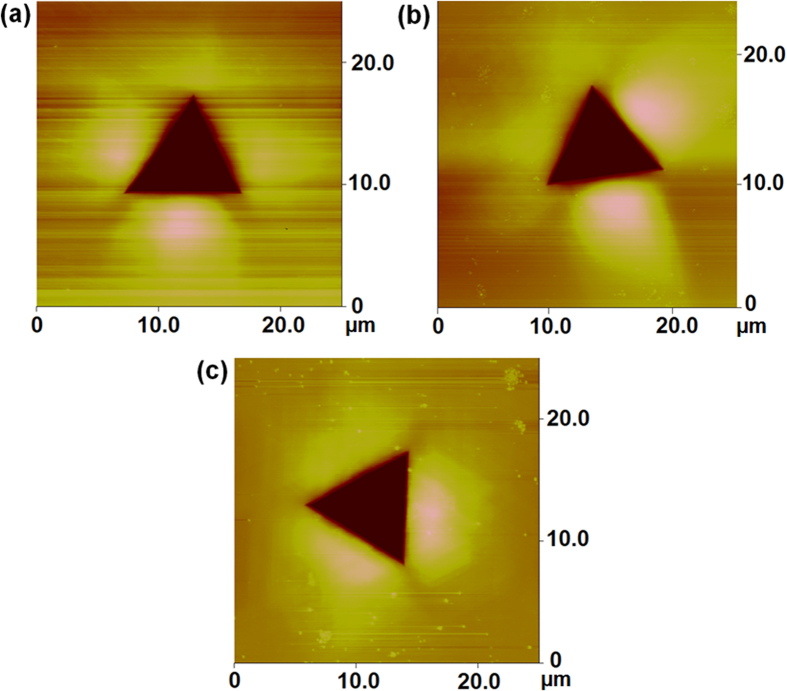
AFM images of the indent impressions made on a single-crystal Al workpiece with a Berkovich indenter (tip radius 200 nm) at different crystallographic orientations: (**a**) (001), (**b**) (101) and (**c**) (111) surfaces.

Figure 6(a–c) shows AFM images of the indent with a Berkovich indenter on three different crystals. The bright colour represents the height profile of the nanoindents (pile-up). The fourfold symmetry of the height profile for the (001) crystal, the twofold symmetry for the (101) crystal and the threefold symmetry for the (111) crystal have been identified according to the height profile. Flom and Komanduri^35^ who performed the indentation tests on the (011) and (111) surfaces of single-crystal Al with a sapphire stylus indenter have made similar observation. Hollatz *et al.*^36^ found a fourfold, twofold, and threefold symmetry of the height profile on the (001), (011), and (111) initial oriented surfaces of single-crystal NiAl respectively. Liu *et al.*^18^ found a fourfold, twofold, and threefold symmetry of the height profile on the (001), (011), and (111) initial oriented surfaces of single-crystals Cu.

**Figure 7:**
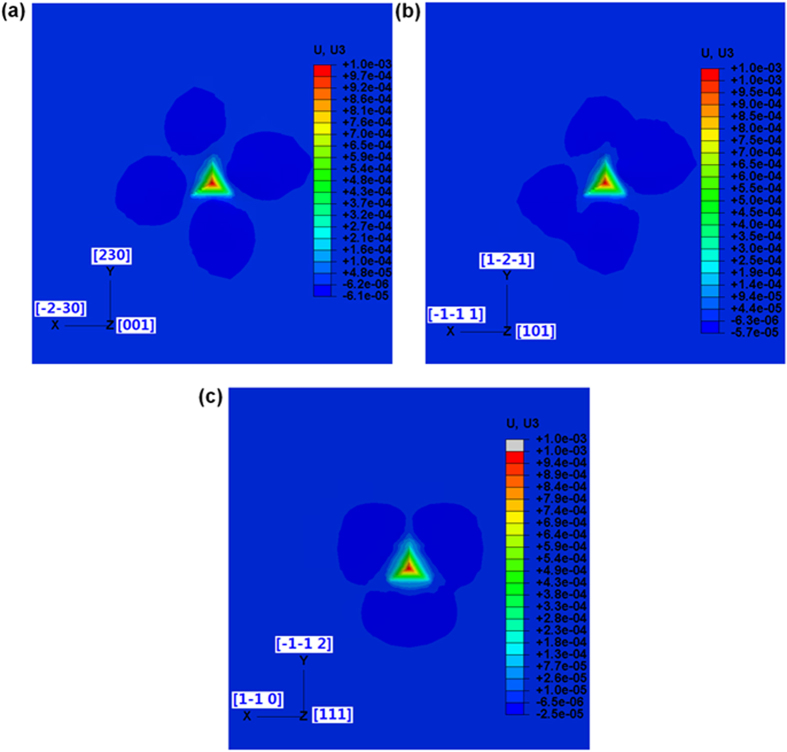
Simulated images of the indent impressions on a single-crystal Al workpiece with a Berkovich indenter (tip radius 200 nm) at different crystallographic orientations: (**a**) (001), (**b**) (101) and (**c**) (111) surfaces.

**Figure 8:**
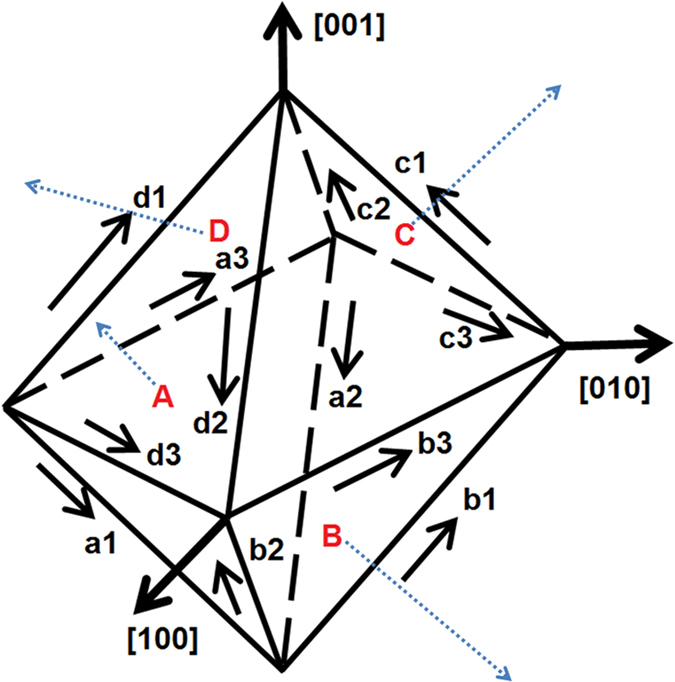
Slip system implemented in the CPFEM model.

Figure 7a–c shows the simulated surface profiles of three surfaces with the different initial orientations after indentation. All the simulated pile-up patterns are consistent with the experimental observations. To the best knowledge of the authors, no satisfactory agreements between numerical and experimental pile-up patterns on single-crystal Al on all three orientations induced by Berkovich indenters have been reported previously^18^. The appearance of the fourfold, twofold, and threefold symmetries on the (001), (011), and (111) initial oriented surfaces of single-crystal Al samples respectively, can be explained by the fact that different slip systems are activated during the indentation of the three different crystals. The 12 slip systems used in the CPFEM model are illustrated in Fig. 8.

With reference to surface orientation (001), Burgers vectors of slip systems a_3_, b_3_, c_3_ and d_3_ shown in Fig. 8 lay perpendicular to the surface normal orientation. Therefore, the slip systems with these Burgers vectors will not be activated. The other four Burgers vectors are [0–11] of slip systems a_1_ and c_1_, [011] of slip systems b_1_ and d_1_, [10–1] of slip systems a_2_ and d_2_, [101] of slip systems b_2_ and c_2_. These four Burgers vectors are oriented with an angle of 45 °C to the surface normal orientation. Therefore, the slip systems including these four Burgers vectors are activated. The normal planes of aforementioned eight slip systems are named A, B, C and D, and they are aligned to top left, bottom right, top right and bottom left pile-up sites. The pile-up at top left, top right, bottom left and bottom right are due to the activations of the a_1_ and a_2_ slip systems, the c_1_ and c_2_ slip systems, the d_1_ and d_2_ slip systems and the b1 and b2 slip systems respectively.

For the surface orientation (101), Burgers vector [101] of slip system b_2_ and c_2_ is parallel to the surface normal orientation and another Burgers vector [10–1] of slip system a_2_ and d_2_ is perpendicular to the surface normal orientation. Therefore, the slip systems including the two Burgers vectors will not be activated. Slip plane A has a normal direction on the right side of (101) surface orientation and slip plane D lay with a normal direction on the left side of the (101) surface. Therefore, the slip systems a_1_ and a_3_ in slip plane A, and the slip systems d_1_ and d_3_ in slip plane D are activated. The other two slip planes B and C have a normal which is perpendicular to the (101) surface orientation respectively, and thus, all the slip systems included in these two slip planes will not be activated. It is obvious that the two pile-up sites at the right are mainly due to the activation of d_1_ and d_3_ slip systems and the other two pile-up sites at the left are due to the activation of a_1_ and a_3_ slip systems.

For the surface orientation (111), the normal of the slip plane A is parallel to surface normal orientation, and thus, the slip systems included in the slip plane A will not be activated. The three Burgers vector of slip systems named b_3_, c_1_ and d_2_ also lie perpendicular to the surface normal orientation, and thus the corresponding slip systems with the three Burgers vectors will not be activated. The other three Burgers vectors are [011] of slip systems b_1_ and d_3_, [101] of slip systems b_2_ and c_2_, and [110] of slip systems c_3_ and d_3_. These three Burgers vectors are oriented at an angle of 60 °C to the surface normal orientation and thus, the slip systems included in these three Burgers vectors are activated. The normal planes of aforementioned three slip systems are named B, C and D, and they lie with normal directions to the three pile-up sites. It can be concluded that the three pile-up peaks are solely due to the activation of b_1_ and b_2_, c_2_ and c_3_ and d_1_ and d_3_ slip systems, respectively.

### Micro-texture evolution

**Figure 9:**
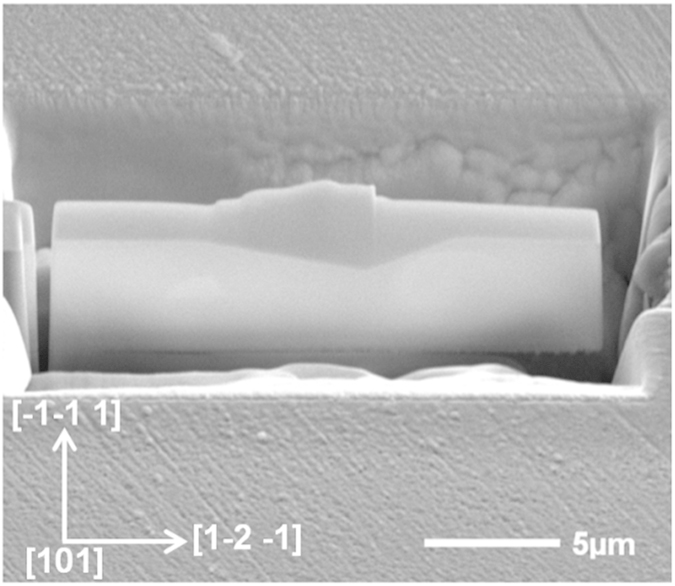
FIB micrographs of a 10 mN indent on the (101) surface before lift-out.

Figure 9 shows the cross-sectional view of an FIB cutting through the centre of a 10 mN indentation impression on the (101) surface before lift-out, using a FIB workstation (XT Nova Nanolab 200). The centre of the impression was marked first in order to obtain a cross-section which proceed right thought the middle of the indent and then a tungsten layer with a thickness of about 500 nm is deposited on the surface to minimize the damage caused by the ion beam.

**Figure 10:**
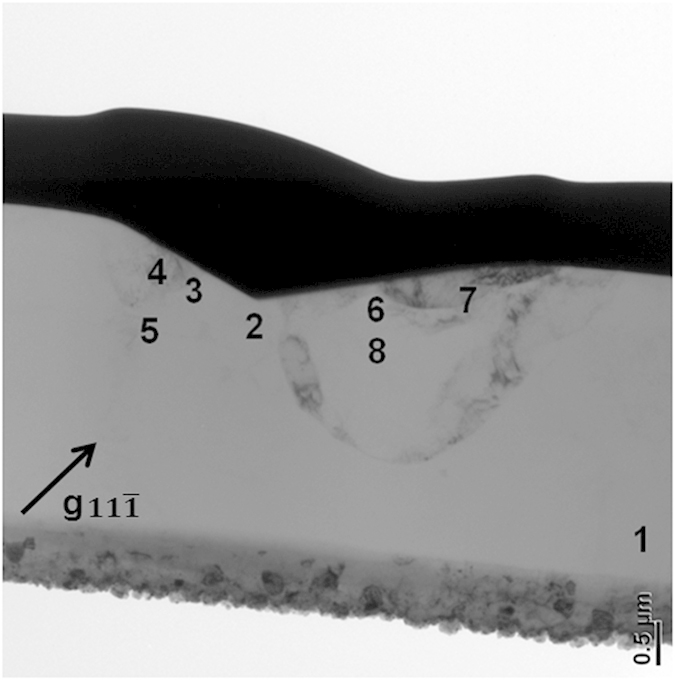
Cross-sectional TEM micrographs of 10 mN indent on the (101) surface, taken with a 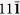 two beam condition.

**Figure 11:**
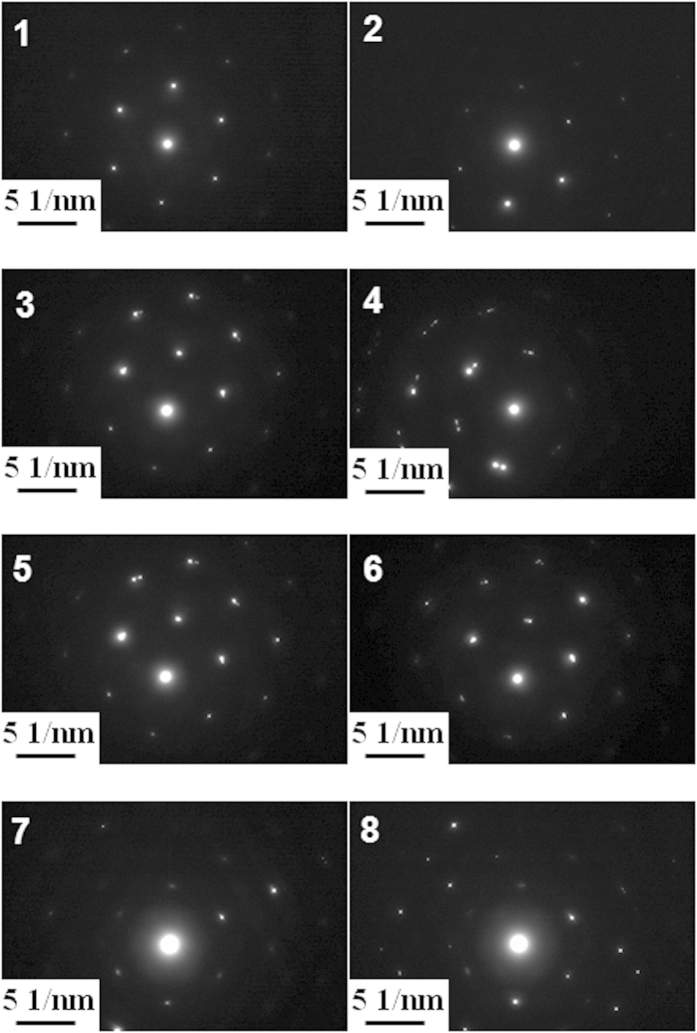
Selected area diffraction patterns which correspond to the regions marked from number 1 to 8 in Figure 10.

Figure 10 presents the bright field image of a 10 mN indenter with number marked from 1 to 8. The numbers marked in the image represent the respective positions where the selected area aperture was positioned. SAED is subsequently performed on the cross-sectional TEM sample at the marked positions (as shown in Fig. 11). Lattice rotation angles along the *x*-axis (RD) can be determined by comparing the SAED patterns of the deformed areas indicated by the number 2 to 8 with the undeformed area marked number 1 (as shown in Table 6).

**Figure 12:**
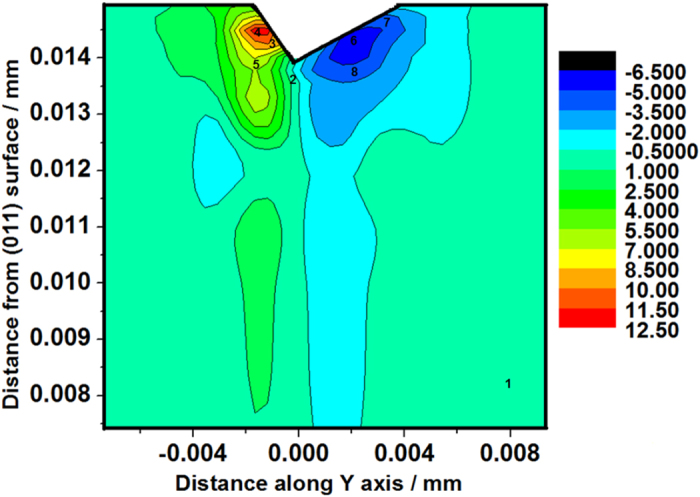
Distributions of rotation angles of the cross-section along Y axis, observed from the 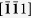 direction.

The positive and negative value represents the counter-clockwise (CCW) rotation and the clockwise (CW) rotation respectively. To compare the simulated rotation of the crystallographic orientation during indentation process with the experimental observation, the misorientation of each node relative to the initial orientation is partitioned into three components representing the rotation angles around the *x*-(RD), *y*-(TD) and *z*-(ND) axes, respectively. The method is proposed by Wert *et al.*^37^. Contour maps of crystalline rotation angles around *x*-axis are shown in Fig. 12. The value of each marked point in Fig. 12 is also listed in Table 6. The results are in a good agreement with the experimental results. Therefore, the CPFEM model developed in this study is able to use the lattice rotation to provide an accurate prediction of the change in micro-texture induced by the nanoindentations. Both the numerical and experimental rotation angles are measured along *x*-axis but are observed in the opposite directions (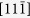 and 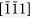) and thus, the experimental value is opposite from the numerical one.

### Relationship between indentation hardness and tensile yield stress

**Figure 13:**
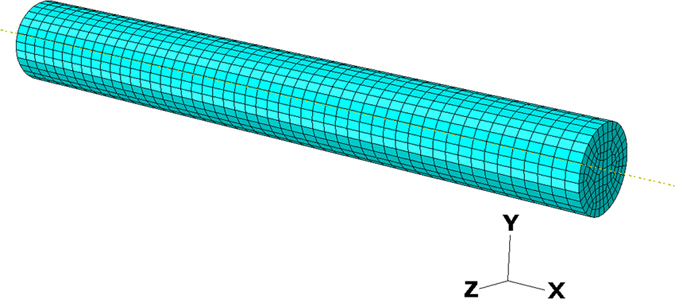
The 3D CPFEM tensile test model.

The simulated tensile test sample is made of a round bar with the length of 4 mm and the diameter of 0.5 mm, as shown in Fig. 13. The total number of elements is 5550. During the simulation the displacement along the *x*-axis at the cross section of *x* = 0 is constrained and a constant speed of 0.001125 mm/s along the *x*-axis has been applied to the cross section of *x* = L, where L is the length of the sample.

**Figure 14:**
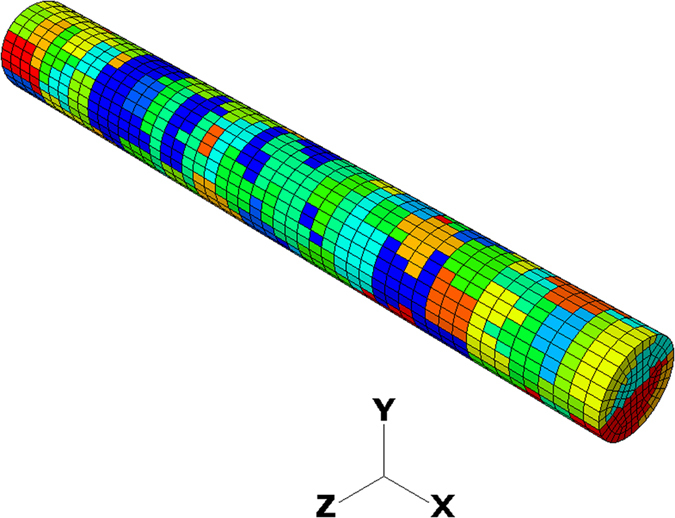
The 3D CPFEM poly-crystal tensile test model.

Currently, the Voronoi diagram is a commonly used method for the construction of polycrystalline material structure^38^. In this study, the 3D Voronori diagram is used to generate a number of three-dimensional cells. Each Voronoi cell corresponds to one seed and the number of seeds can be controlled to determine the average size of the cells. The generated Voronoi cells are then assigned with different crystallographic orientations and implemented into the CPFEM model. In the CPFEM simulation each Voronoi cell represents a virtual grain. The detailed description for constructing poly-crystal CPFEM model has been given in ref. 39. In this study, 135 grains were generated and the grain size is approximately 200 μm as shown in Fig. 14. Different colours are used to indicate the different crystallographic orientations. The grain size used in the present simulation is close to that measured from the annealed commercially pure Al EN AW1050 in ref. 40,41.

**Figure 15:**
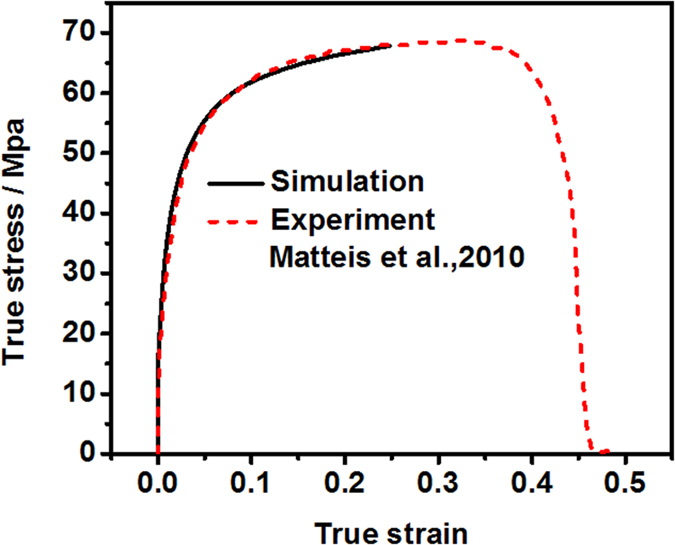
Comparison between the experiment and simulation of tensile test of pure Al^41^

In order to validate the poly-crystal simulation model developed in the present study, the tensile experiment performed on an Al sample by Matteis *et al.*^41^ is simulated. The material used in their tensile test was commercially pure Al alloy EN AW1050 subjected to four annealing cycles. The measured stress-strain curve is shown in Fig. 15.

**Figure 16:**
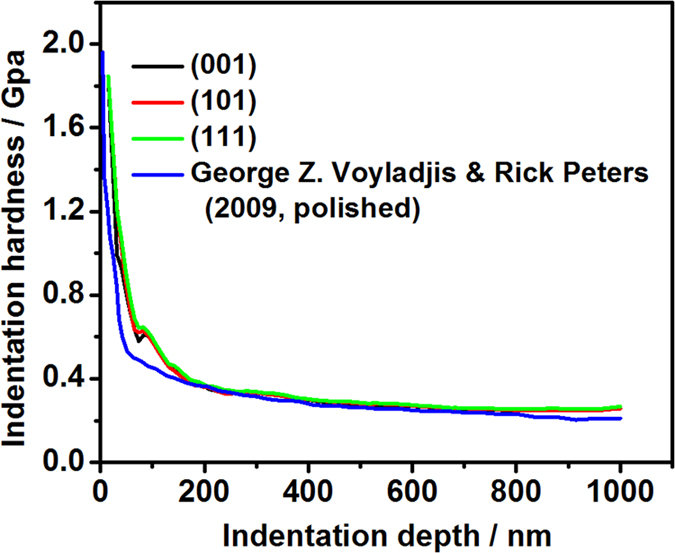
Comparison of hardness-displacement curve derived from the numerical and experimental indentation.

Figure 15 shows comparison between the experiment and simulation of the tensile test of pure Al. The numerical result of the CPFEM model is found to be consistent with the experimental results. In order to validate the numerical hardness-displacement curves, the simulated results have been compared with the experimental data obtained by Voyiadjis and Peter^42^ in 2009. They carried out the nanoindentation tests on the polished surface of a poly-crystal Al sample with 99.9999% purity. As the indents normally locate in a single grain, it can also been treated as a single crystal. Figure 16 shows comparison between experiment and simulation of indentation hardness.

According to Tabor’s research^43^, the relationship between indentation hardness and yield stress of metal material can be expressed as the following equation


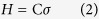


where *σ* is the uniaxial yield stress and *H* is the indentation hardness. The factor C is termed as elastic constraint factor and has a value of approximately 3 for metals with a strain hardening exponent *n* that equates to 0^44^. The yield stress value in Eq. (2) corresponds to the plastic strain that is unique to the hardness test performed, or more specifically, to the geometry of the indenter tip. In the case of the diamond pyramid hardness (DPH) via Vickers, the flow stress corresponds to a plastic strain of 0.08 which is defined as the representative plastic strain^45^. Jayaraman *et al.*^46^,^47^ determined a representative plastic strain of 0.07 and 0.225 for Berkovich and Cube-corner indenters, respectively.

On the other hand, Marcinkowski *et al.*^48^ reported that annealed Fe-Cr alloys exhibited some strain hardening satisfying *H* = 5*σ*. Speich and Warlimont^49^ found that some low carbon martensites and Fe-Ni alloys abided to *H* = 4*σ*.

In the present study, the indentation hardness of single-crystals Al with the different initial orientations were 248 MPa, 249 MPa and 255 MPa, respectively (as shown in Fig. 16). The average hardness is calculated to be 250.7 MPa

It can be obtained from Fig. 15 that the simulated yield stress is about 62 MPa at the true plastic strain of 0.07. Hence, C has a value of 4.04, which is slightly larger than 3, while the strain hardening exponent *n* has a value of 0.237 at the true strain of 0.07. Therefore, the results suggest that the strain hardening has a slight influence on the factor *C*.

## Conclusions

1. A CPFEM model has been established to study the mechanical behavior and micro-texture evolution of single-crystal Al induced by a sharp Berkovich indenter.
2. Both the simulated load-displacement curves and pile-up patterns were analyzed and compared with the results from the experiments. The numerical results are consistent with those from experimental observations for three single-crystal Al samples with different initial orientations.
3. The simulated lattice rotation angles at different places induced by the nanoindentation tests are also in a good agreement with those from the experiment.
4. The 3D poly-crystal tensile model has been established to study the relationship between indentation hardness and yield stress. The simulated results indicate that the elastic constraint factor C is slightly larger than conventional value 3 as a result of the strain hardening.

## Additional Information

**How to cite this article**: Liu, M. *et al.* A combined experimental-numerical approach for determining mechanical properties of aluminum subject to nano-indentation. *Sci. Rep.* **5**, 15072; doi: 10.1038/srep15072 (2015).

## Supplementary Material

Supplementary Information

## Figures and Tables

**Table 1:** Previous studies on deformation-induced lattice rotations during indentation.

Name	Methods	Materials	Research
Lloyd *et al.*^7^	TEM FIB	Single crystal copper	Lattice rotation angles around an axis perpendicular to the [110] zone axis were investigated. It was found that the rotations only occurred in the region immediately below the indent impression. The greatest rotations were quite near the indent tip and the magnitude of rotation angles decreased significantly with the increasing distance from the indent tip along the surface on the shallow side.
Larson *et al.*^12^,^13^,^14^	Non-destructive 3D synchrotron diffraction	Single crystal copper	Larson *et al.* observed a systematic deformation-induced orientation pattern below [111] indents in Cu single crystals. The experimentally observed pattern was characterized by outward rotations at the rim of the indent (tangent zone of the indent) and inward rotations directly below the indent close to the indenter axis.
Wang *et al.*^6^	EBSD	Single crystal copper	Wang *et al.* investigated the dependence of nanoindentation pile-up patterns and of lattice rotations for Cu single crystals with different orientations ([100], [110], and [111]) using a conical indenter. The 2D orientation measurements in this work were conducted around the indents at the surface with a high-resolution EBSD technique but no 3D analysis could be performed at that time.
Rester *et al.*^15^	EBSD FIB	Single crystal copper	It was found that the orientation differences increased with growing indentation depth. The hardness of a material varied with the size of the indent impression and the source size became the dominant effect only for very small impressions.
Zaafarani *et al.*^9^	EBSD FIB	Single crystal copper	The EBSD testings conducted in sets of subsequent 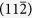 cross-section planes exhibited a pronounced deformation-induced 3D patterning of the lattice rotations below and around the indent.

**Table 2:** Parameters in the constitutive model.

n	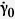 , 1/s	h_0_, MPa	h_*s*_, MPa	τ_1_, MPa	τ_0_, MPa	γ_0_	q
300	0.0001	100	0.01	6.3	6	0.001	1

**Table 3:** Notation of the slip systems for the FCC materials considered in this study.

System	a_1_	a_2_	a_3_	b_1_	b_2_	b_3_	c_1_	c_2_	c_3_	d_1_	d_2_	d_3_
Plane	(111)			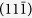			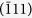			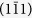		
Direction	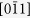	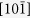	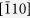	[011]	[101]	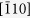	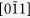	[101]	[110]	[011]	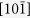	[110]

**Table 4:** Al single crystal properties provided by MaTecK.

Crystalstructure	Productionmethod	Sample size	Orientation	Orientationaccuracy	Roughness ofsurface
FCC	Bridgman	Dia.15 mmThickness 2 mm	(100), (101), (111)	<0.1°	<10 nm

**Table 5:** The Young’s modulus calculated for both simulation and experiment of single-crystal Al samples with different initial orientations.

	Young’s modulus/GPa		
	(001)	(101)	(111)
Experiment	63.18	71.79	75.10
Simulation	63.14	71.56	74.98

**Table 6:** Lattice rotation angles correspond to the regions marked from number 1 to 8 for both simulation and experiment.

No.	1	2	3	4	5	6	7	8
Simulated rotation angles	Reference 0°	1°	10°	12.5°	7°	−5°	−3.5°	−3.5°
Experimental rotation angles	Reference 0°	−1°	−9°	−12°	−7°	5°	4°	3°
