# Supplementary material for: A combined experimental-numerical approach for determining mechanical properties of aluminum subjects to nanoindentation
Source: Sci Rep. 2015 Oct 14;5:15072. doi: 10.1038/srep15072 (PMC4604481; doi:10.1038/srep15072)
Supplement: Supplementary Information [file srep15072-s1.pdf]

Supplementary materials

A combined experimental-numerical approach for determining mechanical properties of aluminum subjects to nanoindentation

Mao Liu[a1], Cheng Lu[a], Kiet Anh Tieu[a], Ching-Tun Peng[a], Charlie Kong[b]

[a] *School of Mechanical, Materials and Mechatronic Engineering, University of Wollongong, Wollongong, NSW 2522, Australia*

[b] *Electron Microscope Unit, The University of New South Wales, Sydney, NSW 2052, Australia*

[1] Corresponding author: Mao L.: ml818@uowmail.edu.au
Tel: +61 0430516926

Constitutive models and finite element simulations

Kinematics of crystalline deformation

The quantitative description of kinematical theory for the mechanics of elastic-plastic deformation of crystals was derived from the early work of Taylor [1] and Hill [2], who indicated that material flows through the crystal lattice via dislocations gliding along the corresponding slip systems. The constitutive models used here followed the earlier work done by Asaro [3], Asaro and Rice [4], and Huynh [5,6].

According to the crystal plasticity theory, the crystalline material under load undergoes crystallographic slip due to dislocation motion on the active slip systems and elastic deformation including stretching and rotating of the crystal lattice [3,5,7-11], which leads to a hypothesis that the crystallographic slip may be supposed to firstly occur from the reference configuration (first configuration) to the intermediate configuration (secondary configuration), followed by the elastic stretching and rotation from the intermediate configuration to the current configuration (third configuration). Therefore, three configurations have been proposed to describe the deformation process. The initial configuration corresponds to the original undeformed state of the element and the current configuration to the deformed state of the element. The secondary configuration can be obtained from the third configuration when relaxing the lattice elastic stretching and rotation. The total deformation gradient (***F***) can be decomposed into two different components, namely $\boldsymbol{F}^*$and $\boldsymbol{F}^{\mathrm{P}}$ as

$$\mathbf{F} = \frac{\partial \mathbf{x}}{\partial \mathbf{X}} = \mathbf{F}^{*} \cdot \mathbf{F}^{\mathrm{P}} \qquad (1)$$

where $\boldsymbol{X}$ represents the position of material points in the reference configuration and $\boldsymbol{x}$ denotes the position of material points in the current configuration. $\boldsymbol{F}$ is the total deformation gradient, $\boldsymbol{F}^{*}$ describes the elastic deformation gradient including the stretching and rotation of the crystal lattice, and $\boldsymbol{F}^{\mathrm{P}}$indicates crystallographic slip on the slip system, which can be written as

$$\mathbf{F}^{\mathrm{P}} = \sum_{\alpha=1}^{\mathrm{N}} \mathbf{F}^{(\alpha)\mathrm{P}} = \sum_{\alpha=1}^{\mathrm{N}} \mathbf{I} + \gamma^{(\alpha)} \left(\mathbf{s}_0^{(\alpha)} \otimes \mathbf{m}_0^{(\alpha)} \right) \qquad (2)$$

where $\boldsymbol{F}^{(\alpha)\mathrm{P}}$ is the contribution of the α-th slip system to $\boldsymbol{F}^{\mathrm{P}}$, $\gamma^{(\alpha)}$ is the shear strain of α-th slip system, ⊗ indicates the dyadic product of two tensors or vectors , $\boldsymbol{I}$ is a second-order unit tensor and N is the number of activated slip systems.

The Green strain tensor can be written as

$$\mathbf{E} = \frac{1}{2}\left(\mathbf{F}^{\mathrm{T}}\mathbf{F} - \mathbf{I}\right) \tag{3}$$

In the initial configuration, the crystal slip system α consist of a slip direction vector $\boldsymbol{s}_0^{(\alpha)}$ lying in a slip plane with normal vector of $\boldsymbol{m}_0^{(\alpha)}$. Both $\boldsymbol{s}_0^{(\alpha)}$ and $\boldsymbol{m}_0^{(\alpha)}$ are unit vectors and should satisfy the orthogonal relationship, namely

$$\mathbf{s}_0^{(\alpha)} \cdot \mathbf{m}_0^{(\alpha)} = 0 \tag{4}$$

Both $\boldsymbol{s}_0^{(\alpha)}$ and $\boldsymbol{m}_0^{(\alpha)}$will not be changed during crystallographic slip from the initial configuration to the intermediate configuration but converted with the lattice when the lattice is stretched and rotated from the intermediate to the current configuration. The slip direction vector $\boldsymbol{s}_0^{(\alpha)}$ can be given in the deformed configuration by

$$\mathbf{s}^{(\alpha)} = \mathbf{F}^{(\alpha)} \cdot \mathbf{s}_0^{(\alpha)} = \mathbf{F}^{*}\left(\sum_{\alpha=1}^{N} \mathbf{I} + \gamma^{(\alpha)}\left(\mathbf{s}_0^{(\alpha)} \otimes \mathbf{m}_0^{(\alpha)}\right)\right)\mathbf{s}_0^{(\alpha)} = \mathbf{F}^{*} \cdot \mathbf{s}_0^{(\alpha)} \tag{5}$$

The normal to the slip plane $\boldsymbol{m}_0^{(\alpha)}$ after deformation can be written as

$$\mathbf{m}^{(\alpha)} = \left[\mathbf{s}^{(\alpha)}\right]^{-\mathbf{1}} = \left(\mathbf{F}^{*} \cdot \mathbf{s}_0^{(\alpha)}\right)^{-1} = \mathbf{m}_0^{(\alpha)} \cdot \mathbf{F}^{*-1} \tag{6}$$

It should be noted that $\boldsymbol{s}^{(\alpha)}$ and $\boldsymbol{m}^{(\alpha)}$ are not in general unit vectors, but remain orthogonal.

$$\mathbf{s}^{(\alpha)} \cdot \mathbf{m}^{(\alpha)} = 0 \tag{7}$$

The current velocity gradient is evaluated from the deformation gradient by

$$\mathbf{L} = \frac{\partial \mathbf{v}}{\partial \mathbf{x}} = \frac{\partial \mathbf{v}}{\partial \mathbf{X}}\frac{\partial \mathbf{X}}{\partial \mathbf{x}} = \dot{\mathbf{F}}\mathbf{F}^{-1} = \mathbf{L}^{*} + \mathbf{L}^{\mathrm{P}} \tag{8}$$

$$\mathbf{L}^{*} = \dot{\mathbf{F}}^{*} \cdot \mathbf{F}^{*-1} \tag{9}$$

$$\mathbf{L}^{\mathrm{P}} = \mathbf{F}^{*} \cdot \dot{\mathbf{F}}^{\mathrm{P}} \cdot \mathbf{F}^{\mathrm{P}-1} \cdot \mathbf{F}^{*-1} \tag{10}$$

where $\boldsymbol{v}$ is the velocity of the material point in the current configuration, $\dot{\boldsymbol{F}}$ represents a time derivative of $\boldsymbol{F}$, $\boldsymbol{L}^*$ is the elastic component of the velocity gradient and $\boldsymbol{L}^{\mathrm{P}}$ is the plastic component of the velocity gradient.

From Eq. (2) $\boldsymbol{L}^{\mathrm{P}}$ can be derived as

$$\mathbf{L}^{\mathrm{P}} = \sum_{\alpha=1}^{\mathrm{N}} \dot{\gamma}^{(\alpha)} \mathbf{s}^{(\alpha)} \otimes \mathbf{m}^{(\alpha)} \quad , \tag{11}$$

and it is related to the shear strain rate $\dot{\gamma}^{(\alpha)}$ caused by the plastic slip in the α-th slip system.

The velocity gradient $\boldsymbol{L}$ can be uniquely decomposed into a symmetric part $\boldsymbol{D}$ (stretching rate tensor) and a skew-symmetric $\boldsymbol{\Omega}$ (spin tensor) part as

$$\mathbf{L} = \mathbf{D} + \mathbf{\Omega} \tag{12}$$

$$\mathbf{D} = \frac{1}{2}\left(\mathbf{L} + \mathbf{L}^{\mathrm{T}}\right) \tag{13}$$

$$\mathbf{\Omega} = \frac{1}{2}\left(\mathbf{L} - \mathbf{L}^{\mathrm{T}}\right) \tag{14}$$

$\boldsymbol{D}$ is also in general called the rate of deformation. Both tensors $\boldsymbol{D}$ and $\boldsymbol{\Omega}$ can also be decomposed into the elastic stretching and lattice rotation component ($\boldsymbol{D}^*$ and $\boldsymbol{\Omega}^*$) and the plastic component ($\boldsymbol{D}^{\mathrm{P}}$ and $\boldsymbol{\Omega}^{\mathrm{P}}$) as follows,

$$\mathbf{D} = \mathbf{D}^* + \mathbf{D}^{\mathrm{P}} \tag{15}$$

$$\mathbf{\Omega} = \mathbf{\Omega}^* + \mathbf{\Omega}^{\mathbf{p}} \tag{16}$$

and then $\boldsymbol{D}^{\mathrm{P}}$ in Eq. (15) can be expressed as

$$\mathbf{D}^{\mathrm{P}} = \frac{1}{2}\left(\mathbf{L}^{\mathrm{P}} + \mathbf{L}^{\mathrm{P}^{\mathrm{T}}}\right) = \sum_{\alpha=1}^{\mathrm{N}} \mathbf{p}^{(\alpha)} \dot{\gamma}^{(\alpha)} \tag{17}$$

where $\boldsymbol{p}(\alpha)$ can be defined as

$$\mathbf{p}^{(\alpha)} = \frac{1}{2}\left(\mathbf{s}^{(\alpha)} \otimes \mathbf{m}^{(\alpha)} + \mathbf{m}^{(\alpha)} \otimes \mathbf{s}^{(\alpha)}\right) \tag{18}$$

Similar to $\boldsymbol{D}^{\mathrm{P}}$, the tensor $\boldsymbol{\Omega}^{\mathrm{P}}$ in Eq. (16) can also be expressed as

$$\mathbf{\Omega}^{\mathrm{p}} = \sum_{\alpha=1}^{\mathrm{N}} \mathbf{w}^{(\alpha)} \dot{\gamma}^{(\alpha)} \tag{19}$$

where $\boldsymbol{w}^{(\alpha)}$ can be defined as

$$\mathbf{w}^{(\alpha)} = \frac{1}{2}\left(\mathbf{s}^{(\alpha)} \otimes \mathbf{m}^{(\alpha)} - \mathbf{m}^{(\alpha)} \otimes \mathbf{s}^{(\alpha)}\right) \tag{20}$$

where $\mathbf{s}^{(\alpha)} \otimes \mathbf{m}^{(\alpha)}$ is Schmid factor. $\boldsymbol{p}^{(\alpha)}$ and $\boldsymbol{w}^{(\alpha)}$ are symmetric and asymmetric part of the Schmid factor, respectively. In the crystalline coordinate system, for an FCC crystal, $\boldsymbol{s}^{(\alpha)}$ and $\boldsymbol{m}^{(\alpha)}$ are given by

$$\mathbf{s}^{(1-3)} = \frac{1}{\sqrt{3}}(1,1,1), \mathbf{s}^{(4-6)} = \frac{1}{\sqrt{3}}(-1,1,1), \mathbf{s}^{(7-9)} = \frac{1}{\sqrt{3}}(1,-1,1), \mathbf{s}^{(10-12)} = \frac{1}{\sqrt{3}}(1,1,-1) \tag{21}$$

$$\mathbf{m}^{(1)} = \frac{1}{\sqrt{2}}(0,-1,1), \mathbf{m}^{(2)} = \frac{1}{\sqrt{2}}(1,0,-1), \mathbf{m}^{(3)} = \frac{1}{\sqrt{2}}(-1,1,0), \mathbf{m}^{(4)} = \frac{1}{\sqrt{2}}(1,0,1),$$

$$\mathbf{m}^{(5)} = \frac{1}{\sqrt{2}}(1,1,0), \mathbf{m}^{(6)} = \frac{1}{\sqrt{2}}(0,-1,1), \mathbf{m}^{(7)} = \frac{1}{\sqrt{2}}(0,1,1), \mathbf{m}^{(8)} = \frac{1}{\sqrt{2}}(1,1,0),$$

$$\mathbf{m}^{(9)} = \frac{1}{\sqrt{2}}(1,0,-1), \mathbf{m}^{(10)} = \frac{1}{\sqrt{2}}(0,1,1), \mathbf{m}^{(11)} = \frac{1}{\sqrt{2}}(1,0,1), \mathbf{m}^{(12)} = \frac{1}{\sqrt{2}}(-1,1,0) \tag{22}$$

The derivatives of Eq. (5) and (6) yield

$$\dot{\mathbf{s}}^{(\alpha)} = \mathbf{L}^{*}\mathbf{s}^{(\alpha)} \tag{23}$$

$$\dot{\mathbf{m}}^{(\alpha)} = -\mathbf{m}^{(\alpha)}\mathbf{L}^{*} \tag{24}$$

By differentiating Eq. (3), the rate of change of Green's Lagrangian strain which refers to the reference configuration, can be written as

$$\dot{\mathbf{E}} = \frac{1}{2}\left(\dot{\mathbf{F}}^{\mathrm{T}}\mathbf{F} + \mathbf{F}^{\mathrm{T}}\dot{\mathbf{F}}\right) = \frac{1}{2}\mathbf{F}^{\mathrm{T}}[(\dot{\mathbf{F}}\mathbf{F}^{-1})^{\mathrm{T}} + \dot{\mathbf{F}}\mathbf{F}^{-1}]\mathbf{F} = \mathbf{F}^{\mathrm{T}}\mathbf{D}\mathbf{F} \tag{25}$$

The polar decomposition theory can be used to decompose the deformation gradient $\boldsymbol{F}$ in Eq. (1) as

$$\mathbf{F} = \mathbf{R}\mathbf{U} \tag{26}$$

where $\boldsymbol{R}$ is the orthogonal rotation tensor and $\boldsymbol{U}$ is the right stretch tensor which is also a positively defined symmetric tensor. They satisfy the condition of

$$\mathbf{R}^{\mathrm{T}} = \mathbf{R}^{-1} \tag{27}$$

$$\mathbf{U} = \mathbf{U}^{\mathrm{T}} \tag{28}$$

Substituting Eq. (26) into Eq. (8) yields

$$\mathbf{L} = \dot{\mathbf{F}}\mathbf{F}^{-1} = \dot{\mathbf{R}}\mathbf{R}^{-1} + \mathbf{R}\dot{\mathbf{U}}\mathbf{U}^{-1}\mathbf{R}^{-1} \quad (29)$$

Namely,

$$\mathbf{L}^{*} = \dot{\mathbf{R}}\mathbf{R}^{-1} \quad (30)$$

$$\mathbf{L}^{\mathrm{p}} = \mathbf{R}\dot{\mathbf{U}}\mathbf{U}^{-1}\mathbf{R}^{-1} \quad (31)$$

$\dot{\boldsymbol{R}}$ is the time derivative of the orthogonal rotation tensor.

Substituting Eq. (16), (30) and (31) into Eq. (14), the asymmetric part $\boldsymbol{\Omega}$ of the velocity gradient can be written as

$$\boldsymbol{\Omega} = \frac{1}{2}\left(\dot{\mathbf{R}}\mathbf{R}^{-1} - \mathbf{R}\dot{\mathbf{R}}^{-1}\right) + \frac{1}{2}\mathbf{R}\left(\dot{\mathbf{U}}\mathbf{U}^{-1} - \mathbf{U}^{-1}\dot{\mathbf{U}}\right)\mathbf{R}^{\mathrm{T}} \quad (32)$$

The derivative of $\mathbf{R}\mathbf{R}^{-1} = \mathbf{I}$ yields

$$\dot{\mathbf{R}}\mathbf{R}^{-1} = -\,\mathbf{R}\dot{\mathbf{R}}^{-1} \quad (33)$$

Supposing $\boldsymbol{U}$ in the reference configuration is a unit tensor, namely

$$\mathbf{U} = \begin{bmatrix} 1 & 0 & 0 \\ 0 & 1 & 0 \\ 0 & 0 & 1 \end{bmatrix} \quad (34)$$

then it is easy to obtain

$$\dot{\mathbf{U}}\mathbf{U}^{-1} = \quad \dot{\mathbf{U}}\mathbf{U}^{-1} \quad (35)$$

Therefore, $\boldsymbol{\Omega}$ in Eqs. (14) and (16) can finally be expressed as

$$\boldsymbol{\Omega} = \dot{\mathbf{R}}\mathbf{R}^{-1} \quad (36)$$

and then $\boldsymbol{R}$ can be expressed by

$$\mathbf{R} = \mathbf{I} - \left(\mathbf{I} + \frac{1}{2}\boldsymbol{\Omega}\Delta\mathrm{t}\right)\left(\mathbf{I} - \frac{1}{2}\boldsymbol{\Omega}\Delta\mathrm{t}\right)^{-1} \quad (37)$$

where, Δt is the time increment. Accordingly, Eq. (32) can also be expressed as

$$\boldsymbol{\Omega} = \frac{2}{\Delta\mathrm{t}}(\mathbf{R} - \mathbf{I})(\mathbf{R} - \mathbf{I})^{-1} \quad (38)$$

Constitutive law

Assuming that $\boldsymbol{t}_0$ is the Kirchhoff stress[3] in the reference configuration at the time $t+\Delta t$, it is also the Kirchhoff stress in the current configuration at the time t. According to the aforementioned description, deformation occurs first by crystallographic slip from the reference configuration to the intermediate configuration, and then the lattice stretching and rotation from the intermediate configuration to the current configuration. It is assumed that the change in stress caused by the slip and lattice stretching is $\dot{\boldsymbol{t}}_0\Delta t$, where $\dot{\boldsymbol{t}}_0$ is the stress rate in the reference configuration. The stress ($\boldsymbol{t}_0 + \dot{\boldsymbol{t}}_0\Delta t$) will be rotated to the current configuration. The rotation tensor is $\boldsymbol{R}$. The Kirchhoff stress[3] $\boldsymbol{t}$ in the current configuration can be written as

$$\mathbf{t} = \mathbf{R}(\mathbf{t_0} + \dot{\mathbf{t}}_\mathbf{0}\Delta \mathrm{t})\mathbf{R}^\mathrm{T} \tag{39}$$

Taking the time derivative of Eq. (39) yields

$$\dot{\mathbf{t}} = \mathbf{R}\dot{\mathbf{t}}_0\mathbf{R}^\mathrm{T} + \dot{\mathbf{R}}(\mathbf{t}_0 + \dot{\mathbf{t}}_0\Delta \mathrm{t})\mathbf{R}^\mathrm{T} + \mathbf{R}(\mathbf{t}_0 + \dot{\mathbf{t}}_0\Delta \mathrm{t})\dot{\mathbf{R}}^\mathrm{T} = \mathbf{R}\dot{\mathbf{t}}_0\mathbf{R}^\mathrm{T} + \boldsymbol{\Omega}\mathbf{t} - \mathbf{t}\boldsymbol{\Omega} \tag{40}$$

where $\dot{\boldsymbol{t}}$ is the material rate of Kirchhoff stress[3]. $\boldsymbol{R}\dot{\boldsymbol{t}}_\mathbf{0}\boldsymbol{R}^\mathrm{T}$ is defined as the Jaumann rate of Kirchhoff stress[3] ($\overset{\nabla}{\boldsymbol{t}}$) on axes that rotate with the material. Therefore, Eq. (40) can be written as

$$\overset{\nabla}{\mathbf{t}} = \dot{\mathbf{t}} - \boldsymbol{\Omega}\mathbf{t} + \mathbf{t}\boldsymbol{\Omega} \tag{41}$$

If deformation from the intermediate to the current configuration alone is taken into account then Eq. (41) can be rewritten as

$$\overset{\nabla}{\mathbf{t}^*} = \dot{\mathbf{t}} - \boldsymbol{\Omega}^*\mathbf{t} + \mathbf{t}\boldsymbol{\Omega}^* = \mathbf{R}\dot{\mathbf{t}}_1^*\mathbf{R}^\mathrm{T} \tag{42}$$

where $\overset{\nabla}{\boldsymbol{t}^*}$ is the Jaumann rate of Kirchhoff stress[3] on axes that rotate with the lattice and $\dot{\boldsymbol{t}}_\mathbf{1}^*$ is the rate of the Kirchhoff stress[3] in the intermediate configuration.

The difference between Eqs. (42) and (41) can be obtained as

$$\overset{\nabla}{\mathbf{t}^*} - \overset{\nabla}{\mathbf{t}} = \sum_{\alpha=1}^{\mathrm{N}} \boldsymbol{\beta}^{(\alpha)}\dot{\gamma}^{(\alpha)} \tag{43}$$

where $\boldsymbol{\beta}^{(\alpha)}$ is defined by

$$\boldsymbol{\beta}^{(\alpha)} = \mathbf{w}^{(\alpha)}\mathbf{t} - \mathbf{t}\mathbf{w}^{(\alpha)} \tag{44}$$

The lattice is elastically stretched along the lattice axis. The lattice stretching can be described in the lattice coordinate system by

$$\dot{\mathbf{t}}_{\mathrm{L}} = \mathbf{C}_0 : \mathbf{D}_{\mathrm{L}} \tag{45}$$

where $\dot{\boldsymbol{t}}_{\mathrm{L}}$ is the material rate of the Kirchhoff stress in the lattice coordinate system, $\boldsymbol{D}_{\mathrm{L}}$ is the rate of the elastic stretching in the lattice coordinate system, $\boldsymbol{C}_0$ is the elastic moduli tensor.

Provided the rotation tensor between the lattice coordinate system and the current configuration is $\boldsymbol{R}_{\mathrm{L}}$, the elastic deformation rate $\boldsymbol{D}^*$ in the current configuration can be linked to $\boldsymbol{D}_{\mathrm{L}}$ by the following equation

$$\mathbf{D}^* = \mathbf{R}_{\mathrm{L}}\mathbf{D}_{\mathrm{L}}\mathbf{R}_{\mathrm{L}}^{\mathrm{T}} \tag{46}$$

The rate of Kirchhoff stress in the intermediate configuration can be expressed by

$$\dot{\mathbf{t}}_1^* = \mathbf{R}^{\mathrm{T}}\mathbf{R}_{\mathrm{L}}\dot{\mathbf{t}}_{\mathrm{L}}\mathbf{R}_{\mathrm{L}}^{\mathrm{T}}\mathbf{R} \tag{47}$$

Therefore, according to Eqs. (27) and (42), the Jaumann rate $\overset{\nabla}{\boldsymbol{t}^*}$ in Eq. (42) can be written as

$$\overset{\nabla}{\mathbf{t}^*} = \mathbf{R}_{\mathrm{L}}\dot{\mathbf{t}}_{\mathrm{L}}\mathbf{R}_{\mathrm{L}}^{\mathrm{T}} \tag{48}$$

Eqs. (46) and (48) can be rewritten as

$$\mathbf{D}_{\mathrm{L}} = \left(\mathbf{R}_{\mathrm{L}}^{\mathrm{T}}\otimes\mathbf{R}_{\mathrm{L}}^{\mathrm{T}}\right) : \mathbf{D}^* \tag{49}$$

$$\dot{\mathbf{t}}_{\mathrm{L}} = \left(\mathbf{R}_{\mathrm{L}}^{\mathrm{T}}\otimes\mathbf{R}_{\mathrm{L}}^{\mathrm{T}}\right) : \overset{\nabla}{\mathbf{t}^*} \tag{50}$$

substituting Eqs. (49) and (50) into Eq. (45) yields

$$\overset{\nabla}{\mathbf{t}^*} = \mathbf{C} : \mathbf{D}^* \tag{51}$$

$$\mathbf{C} = (\mathbf{R}_{\mathrm{L}}\otimes\mathbf{R}_{\mathrm{L}}) \cdot \mathbf{C}_0 \cdot \left(\mathbf{R}_{\mathrm{L}}^{\mathrm{T}}\otimes\mathbf{R}_{\mathrm{L}}^{\mathrm{T}}\right) \tag{52}$$

where $\boldsymbol{C}$ is the tensor of the elastic modulus defined by

$$[\mathbf{C}] = \begin{bmatrix} \mathbf{c}_{11} & \mathbf{c}_{12} & \mathbf{c}_{13} & 0 & 0 & 0 \\ \mathbf{c}_{12} & \mathbf{c}_{22} & \mathbf{c}_{23} & 0 & 0 & 0 \\ \mathbf{c}_{13} & \mathbf{c}_{23} & \mathbf{c}_{33} & 0 & 0 & 0 \\ 0 & 0 & 0 & \mathbf{c}_{44} & 0 & 0 \\ 0 & 0 & 0 & 0 & \mathbf{c}_{55} & 0 \\ 0 & 0 & 0 & 0 & 0 & \mathbf{c}_{66} \end{bmatrix} \tag{53}$$

For an FCC crystal, just three parameters c_{11} , c_{12}, c_{44} are needed.

Substituting Eqs. (17) and (51) into Eq. (43), we can have

$$\overset{\nabla}{\mathbf{t}} = \mathbf{C} : \mathbf{D} - \sum_{\alpha=1}^{N} \left(\mathbf{C} : \mathbf{P}^{(\alpha)} + \boldsymbol{\beta}^{(\alpha)}\right)\dot{\gamma}^{(\alpha)} \tag{54}$$

It was assumed that crystallographic slip is the only plastic deformation mechanism. The resolved shear stress on each slip system can be used as the vital variable to evaluate plastic flow. The resolved shear stress $\tau^{(\alpha)}$ can be calculated by

$$\tau^{(\alpha)} = \mathbf{P}^{(\alpha)} : \mathbf{t} \tag{55}$$

Taking the time derivative gives

$$\dot{\tau}^{(\alpha)} = \left(\mathbf{C} : \mathbf{P}^{(\alpha)} + \boldsymbol{\beta}^{(\alpha)}\right) : \left(\mathbf{D} - \sum_{\beta=1}^{N} \mathbf{P}^{(\beta)}\dot{\gamma}^{(\beta)}\right) \tag{56}$$

The relationship of the Cauchy stress σ and the Kirchhoff stress is

$$\mathbf{t} = \mathrm{J}\boldsymbol{\sigma} \tag{57}$$

And J is defined as

$$\mathrm{J} = \frac{1}{|\mathbf{F}|} \tag{58}$$

Therefore, the constitutive law based on Cauchy stress can be expressed as

$$\boldsymbol{\sigma} = \mathbf{R}\left(\boldsymbol{\sigma}_0\left(1 + \mathrm{tr}(\mathbf{D})\right)\right)\mathbf{R}^{\mathrm{T}} + \overset{\nabla}{\boldsymbol{\sigma}_0}\Delta \mathrm{t} \tag{59}$$

$$\overset{\nabla}{\boldsymbol{\sigma}} = \mathbf{C} : \mathbf{D} - \mathrm{tr}(\mathbf{D})\boldsymbol{\sigma} - \sum_{\alpha=1}^{N} \left(\mathbf{C} : \mathbf{p}^{(\alpha)} + \boldsymbol{\Omega}^{(\alpha)}\boldsymbol{\sigma} - \boldsymbol{\sigma}\boldsymbol{\Omega}^{(\alpha)}\right)\dot{\gamma}^{(\alpha)} \tag{60}$$

$$\dot{\tau}^{(\alpha)} = \left(\mathbf{C} : \mathbf{p}^{(\alpha)} + \mathbf{W}^{(\alpha)}\boldsymbol{\sigma} - \boldsymbol{\sigma}\mathbf{W}^{(\alpha)}\right) : \left(\mathbf{D} - \sum_{\beta=1}^{N} \mathbf{p}^{(\beta)}\dot{\gamma}^{(\beta)}\right) \tag{61}$$

where $\overset{\nabla}{\boldsymbol{\sigma}}$ is the Jaumann rate of Cauchy stress on axes rotating with the material.

Rate-dependent hardening model

The crystal plasticity constitutive model used in this study follows the line described by Asaro [3] and it was incorporated into the implicit finite element code ABAQUS/Standard through the

user material subroutine (UMAT). The noticeable functions of UMAT are to provide the material with the Jacobian matrix for the constitutive model, and to update the stresses and the solution dependent state variables. In this study, we adopted the UMAT framework initially developed by Huang [12] and used Bassani and Wu's [13] formulation as the hardening model which has been described in Ref.[14,15]. The formulations of the rate-dependent hardening model used in the present study can be expressed as

$$\dot{\gamma}^{(\alpha)} = \dot{\gamma}_0^{(\alpha)} \mathrm{sgn}\left(\tau^{(\alpha)}\right) \left|\frac{\tau^{(\alpha)}}{\tau_c^{(\alpha)}}\right|^{n} \tag{62}$$

and

$$\mathrm{sgn}(x) = \begin{cases} -1, & x < 1 \\ 1, & x \geq 1 \end{cases} \tag{63}$$

where $\dot{\gamma}_0^{(\alpha)}$ is the reference (initial) value of the shear strain rate, which is a constant for all the slip systems. n is the strain rate sensitive exponent. Both $\dot{\gamma}_0^{(\alpha)}$ and n are the material parameters. $\tau_c^{(\alpha)}$ is the critical resolved shear stress of the slip system α, which represents the strength of the material.

The rate of change of the critical resolved shear stress is expressed by [2]

$$\dot{\tau}_c{}^{(\alpha)} = \sum_{\beta=1}^{N} f_{\alpha\beta} h_{\alpha\beta}\, \dot{\gamma}^{(\beta)} \tag{64}$$

$$h_{\alpha\alpha} = \left[(h_0 - h_s) sech^2\left(\frac{(h_0 - h_s)\gamma^{(\alpha)}}{\tau_1 - \tau_0}\right) + h_s\right]\left[1 + \sum_{\substack{\beta=1 \\ \beta\neq\alpha}}^{N} f_{\alpha\beta} tanh\left(\frac{\gamma^{(\beta)}}{\gamma_0}\right)\right], \quad for\ \alpha = \beta \tag{65a}$$

$$h_{\alpha\beta} = q h_{\alpha\alpha}, \qquad for\ \alpha \neq \beta \tag{66b}$$

where $h_{\alpha\beta}$ is the hardening modulus including the self-hardening of each system (α=β) and latent hardening (α≠β), q is the latent hardening parameter, γ_0 is the reference value of slip, γ is the shear strain, τ_0 is the initial critical resolved shear stress, τ_1 is the breakthrough shear stress where large plastic flow initiates, h_0 is the hardening modulus just after initial yield, h_s is the

hardening modulus during easy glide and $f_{\alpha\beta}$ is the magnitude of the strength of a particular slip interaction between two slip systems α and β. The factor $f_{\alpha\beta}$ depends on the geometric relation between two slip systems. There are five constants for $f_{\alpha\beta}$, namely α_1(no junction), α_2 (Hirth lock), α_3 (coplanar junction), α_4 (glissile junction) and α_5 (sessile junction).

References

1 Taylor, G. I. Plastic strain in metals. *journal of the Institute of Metals* **62**, 307-324 (1938).

2 Hill, R. Generalized constitutive relations for incremental deformation of metal crystals by multislip. *Journal of the Mechanics and Physics of Solids* **14**, 95-102 (1966).

3 Asaro, R. J. Crystal Plasticity. *J Appl Mech-T Asme* **50**, 921-934 (1983).

4 Asaro, R. J. & Rice, J. R. Strain localization in ductile single crystals. *Journal of the Mechanics and Physics of Solids* **25**, 309-338 (1977).

5 Si, L. Y., Lu, C., Huynh, N. N., Tieu, A. K. & Liu, X. H. Simulation of rolling behaviour of cubic oriented al single crystal with crystal plasticity FEM. *Journal of Materials Processing Technology* **201**, 79-84 (2008).

6 Huynh, N. N. A modelling of Microstructure Evolution and Crack Opening in FCC Materials under Tension. *PhD Dissertation, University of Wollongong, Australia* (2009).

7 Asaro, R. J. & Rice, J. R. Strain Localization in Ductile Single-Crystals. *Journal of the Mechanics and Physics of Solids* **25**, 309-338 (1977).

8 Huynh, N. N. *A modelling of Microstructure Evolution and Crack Opening in FCC Materials under Tension* PhD thesis, University of Wollongong, (2009).

9 Peirce, D., Asaro, R. J. & Needleman, A. An Analysis of Nonuniform and Localized Deformation in Ductile Single-Crystals. *Acta Metall Mater* **30**, 1087-1119 (1982).

10 Asaro, R. J. Micromechanics of Crystals and Polycrystals. *Adv Appl Mech* **23**, 1-115 (1983).

11 Asaro, R. J. & Needleman, A. Overview .42. Texture Development and Strain-Hardening in Rate Dependent Polycrystals. *Acta Metall Mater* **33**, 923-953 (1985).

12 Huang, Y. G. A user-material subroutine incorporating single crystal plasticity in the ABAQUS finite element program. (Harvard University, 1991).

13 Bassani, J. L. & Wu, T. Y. Latent Hardening in Single-Crystals .2. Analytical Characterization and Predictions. *P Roy Soc Lond a Mat* **435**, 21-41 (1991).

14 Lu, C. *et al.* Crystal plasticity modeling of texture evolution and heterogeneity in equal channel angular pressing of aluminum single crystal. *Acta Materialia* **59**, 3581-3592 (2011).

15 Liu, M., Lu, C., Tieu, K. & Yu, H. Numerical comparison between Berkovich and conical nano-indentations: Mechanical behaviour and micro-texture evolution. *Materials Science and Engineering: A* **619**, 57-65 (2014).
